# Implantation

**Published:** 1887-05

**Authors:** 


ARTICLE IV.

### IMPLANTATION—BOTH SIDES.

[Drs. Weld, Clowes, and Allen in Odontological Society—Hill, Brockway, and Perry reply.]

Dr. Weld. I presume Dr. Younger would feel disappointed if he did not meet with some adverse criticism to stimulate him; therefore, I think he will excuse me if I criticise the practice somewhat. I have had some experience in the replantation and transplantation of teeth; have been through the same enthusiasm that Dr. Younger is going through now, and I think he will finally go through the same disappointment which I did. I have replanted between seventy-five and eighty teeth, beginning six or seven years ago; and I do not believe there is now more than one of these eighty teeth in the mouth. I am therefore obliged to condemn the practice, except in places where it is absolutely necessary, in young persons. My knowledge of implantation, from the time John Hunter made a human tooth grow in a cock's comb, and my views of the physiological doctrine of repair, which differ materially from Dr. Younger's, lead me to say that, in my opinion, the practice of implantation is barbarous and worthless. I have read Dr. Younger's pamphet on the "Implantation of Teeth and Pericemental Life." It is pretty hard to criticise it, for on the first page the gentleman states that it is not free from crudities. Now, a crudity is something raw, something undigested; and *that* the paper surely is. It contains many statements which are unfair and misleading, and many that are purely suppositions and unsupported by a single fact. The paper throughout is sadly deficient in all points relating to the physiological doctrine of repair, so far as I can judge. This practice which has been recommended to us will be discarded precisely as replantation and transplantation were discarded, and for the same reason. Three weeks from to-night I am to read a paper before the First District Dental Society on the subject, and shall do what I can to help Dr. Younger digest a subject which he admits in his paper is undigested, and I will give him the opportunity at that time to criticise me as I have criticised him to-night.

Dr. Younger. I am sorry to say that I shall be three thousand miles away at that time. When I go back to San Francisco it is my intention to investigate this subject with the microscope, and see what that has to say about it. I am not dubious about the result.

Dr. Jarvie. I would like to ask Dr. Weld if the failures of his cases of replantation were not the result of diseased conditions about the roots—alveolar abscess, for instance.

Dr. Weld. Just the contrary. I became enamored with replantation, and the idea of crowning roots in this connection occurred to me, and I had special porcelain crowns made for the purpose. Probably fifty per cent. of all the teeth which I replanted were healthy teeth and had healthy environments.

Dr. J. W. Clowes. The unique operations shown us this evening are beautiful to behold, and, as illustrated by the presence of an intelligent and refined lady, are calculated to produce a favorable impression. They remind me of some which I saw several years ago when Dr. Weld was an enthusiast in replanting and transplanting teeth. At that time I was invited by him to see the wondrous results of his labors in this peculiar field. Cases were shown of work done months before, and apparently with success, Others were more recent and full of promise, while others still were accomplished in my presence with undoubting faith in their stability. I spent a forenoon in witnessing these results, and formed my opinion of their, probable value. A month later, at one of our society meetings, as I entered the room Dr. Weld came up to me, still full of enthusiasm, and said, "In less than five years this will be the universal practice in our profession." "What practice will be universal?" I inquired. "Why, extracting teeth before filling them, inserting crowns on their roots, etc." I replied that I hoped not, for what I had seen at his office had affected me so unfavorably that the practice of those operations was something terrible to contemplate; that I went home after visiting him and scarcely slept at night from thinking of the mistake he had made,—the mischief he was working by his hands, and the influence he might have on others in learing them astray. "Ah," he said, "you make me feel badly." Through courtesy I had refrained from expressing any opinions before, but approached in this way, I could not withhold it any longer. Five years later I met Dr. Weld, and inquired if he still practiced those operations. "Oh, no," he replied; "I have long since given them up; they were all miserable failures." I said, "that is just what I expected they would be." Ever since then I have had a high opinion of Dr. Weld, and consider him a sensible young man,—because he not only had the wisdom to discover what was wrong, but sense and pluck enough to take the right side when convinced of his error.

Dr. Allan. We can look at Dr. Younger's paper from two standpoints. One is the stand-point of fact; and the facts that we have seen to-night show that teeth can be implanted and made for a certain length of time to keep their place. Further than that I would not go with Dr. Younger. From a physiological view I think the practice will prove erroneous and misleading. I think Dr. Younger's description or theory of the way in which implanted teeth are retained in place is open to criticism. I am not aware that any distinct membrane has ever been discovered as lining the lacunæ of the bone; certainly such a membrane has never been demonstrated as lining the canaliculi of the bone; and there cannot be, therefore, even a minute portion of the pericementum tissue in the new socket that is made. But there can be a plasma thrown out, which will form an artificial cement, as it were, around the implanted tooth, and for a time hold it in place. But, just as certain as the laws of life and death prevail, there is incompatibility between living tissue and dead tissue, and the time will come when the living tissue will throw out the dead, and the implantation will be a failure. There can be no living union between dead tissue and living tissue. The teeth which Dr. Younger extracts and implants into new sockets in the bone have, he says, a pericemental membrane. Underneath that is the cement, which has been out of the mouth for some length of time. Dr. Younger cannot maintain that that is living tissue. The cementum is dead and the dentine is dead. But granting, for the sake of argument, that the pericementum covering is not absolutely dead, the preparation which Dr. Younger applies—the bichloride of mercury—would certainly destroy any remaining life, as it acts directly on the protoplasm. This union that we see is most beautiful, but it is not a living one. Such operations are doubtful at best, and always dangerous.

Dr. O. E. Hill. Dr. Allan has stated that there cannot be union between living and dead tissue. He also states that if there had been any life in the pericementum it could not possibly survive the course of treatment to which Dr. Younger subjected it. I was anxious to learn whether there was any attachment between the gum and the implanted tooth which we have had the privilege of seeing this evening, and I gave Dr. Atkinson my pen-knife and asked him to try to ascertain. He did so, and reported that the gum was thoroughly attached to the tooth. The knife-blade was very thin, and he used it very carefully, yet the lady winced and the blood started in trying to separate the tooth from the gum. Does not this prove either that the pericementum of that tooth possessed life when implanted or that living and dead tissue do unite? I have seen Dr. Younger operate several times, and have each time been surprised to see how carefully, tenderly, and rapidly the teeth were implanted. I wish to call attention to one point in Dr. Younger's practice which seems singular to me. When he implants a tooth he expects it to become, within a week or so, a line or two shorter than it was when it was inserted; he makes allowance for that, and it does become shorter.

Dr. A. H. Brockway. I do not propose to spend time in discussing the question as to whether there is or can be a union between dead tissue and living tissue, but I wish to make this point: Here is an operatton performed which is an apparent success. We have statements that similar operations have been made where success has obtained for as long as twelve years or more. The average of success undoubtedly has been several years, even under the unfavorable conditions in which tne operation has been many times performed. Now gentlemen get up and say that this operation can only be a failure. Failure is a relative term. We are all in the habit of speaking of our success in filling teeth. What is the average duration of our fine fillings? I fancy most of us would be ashamed to say, if we knew. It seems to me an operation that promises to endure successfully for at least several years, if properly done, cannot justly be considered a failure. Suppose the teeth are expelled after a term of years. What is to prevent the operation being repeated? Viewed in this way, it seems to me we have practically success in this operation.

Dr. Perry. I think the operation we have seen so well illustrated this evening may be called successful if the teeth last only three or five years. At all events, it would be hard to convince the patient that the operation is not a success. Whatever may be our learned theories, we must not shut our eyes to the accomplished fact which is before us in this lady's mouth. I have seen enough of Dr. Younger's work during the last ten years to satisfy me that, if any one can make a success of these operations, the delicacy and skill of his manipulations will enable him to do it. On general principles, I should be ready to sustain him in his practice of opening the apex, removing the pulp, and filling the root from that end. In replacing teeth I have never managed them in that way, but the idea, which I confess is new to me, is one I should favor. Treated in this way, there could be no chance of leaving any portion of the pulp at the apex to give rise to future trouble.—*Items of Interest.*

